# Attention-deficit/hyperactivity disorder (ADHD) in adult obese patients referred to bariatric surgery

**DOI:** 10.1192/j.eurpsy.2022.977

**Published:** 2022-09-01

**Authors:** G.E. Brancati, M. Barbuti, F. Weiss, A. Calderone, F. Santini, G. Perugi

**Affiliations:** 1 University of Pisa, Department Of Clinical And Experimental Medicine, Pisa, Italy; 2 University of Pisa, Clinical And Experimental Medicine, Pisa, Italy

**Keywords:** Eating Disorders, obesity, adhd, bariatric surgery

## Abstract

**Introduction:**

Attention-deficit/hyperactivity disorder (ADHD) is a neurodevelopmental condition characterized by symptoms of inattention, hyperactivity, and impulsivity, which only rarely remits in adulthood^[1]^. A positive association between ADHD and obesity has been repeatedly observed, especially in adult samples^[2]^. However, only a few studies investigated the prevalence and correlates of ADHD in obese patients seeking bariatric treatment^[3,4]^.

**Objectives:**

Our study was aimed to examine the prevalence of probable ADHD comorbidity in a sample of obese patients referred for bariatric surgery. Secondly, we sought to characterize differences in eating behaviour between obese subjects with and without probable ADHD.

**Methods:**

The study sample was composed of 110 adult obese patients (BMI ≥ 30 kg/m2) consecutively referred for bariatric surgery to the Obesity Center of the Endocrinology Unit in Pisa University Hospital between November 2010 and May 2012. Probable ADHD was identified using a recently developed screening scale based on items selected from Symptom Check‐List‐90‐R (SCL-90-R)^[5]^. The extent of binge-eating/purging and night-eating behaviours were respectively estimated using the Bulimic Investigatory Test, Edinburgh (BITE)^[6]^ and the Night-eating Questionnaire (NEQ)^[7]^. Wilcoxon test was used for statistical comparisons, with a significance level of p<0.05 set for all tests.

**Results:**

Probable ADHD was found in 14 subjects (12.7%, 95%CI=7.1-20.4%). Patients with probable ADHD showed significantly higher BITE symptom score (20.4±9.3 vs. 12.1±7.5, r=0.31, p=0.001) and NEQ total score (16.1±9.2 *vs.* 9.5±3.9, r=0.27, p=0.005).

**Conclusions:**

ADHD is a relatively common comorbidity in obese patients seeking bariatric treatment, which is positively associated with disordered eating habits, such as binge-eating/purging and night-eating behaviours.

**Disclosure:**

No significant relationships.

